# Reductions in non-medical prescription opioid use among adults in Ontario, Canada: are recent policy interventions working?

**DOI:** 10.1186/1747-597X-8-7

**Published:** 2013-02-14

**Authors:** Benedikt Fischer, Anca Ialomiteanu, Paul Kurdyak, Robert E Mann, Jürgen Rehm

**Affiliations:** 1 Centre for Applied Research in Mental Health and Addictions (CARMHA), Faculty of Health Sciences, Simon Fraser University, 2400-515 W Hastings St., V6B 5K3, Vancouver, BC, Canada; 2 Social and Epidemiological Research Department, Centre for Addiction and Mental Health, Toronto, ON, Canada; 3 Department of Psychiatry, University of Toronto, Toronto, Canada; 4 Dalla Lana School of Public Health, University of Toronto, Toronto, Canada; 5 Technische Universitaet Dresden, Dresden, Germany

**Keywords:** Prescription opioids, Non-medical use, Policy, Interventions, Prescription monitoring, Population health, Canada

## Abstract

**Background:**

Non-medical prescription opioid use (NMPOU) and prescription opioid (PO) related harms have become major substance use and public health problems in North America, the region with the world’s highest PO use levels. In Ontario, Canada’s most populous province, NMPOU rates, PO-related treatment admissions and accidental mortality have risen sharply in recent years. A series of recent policy interventions from governmental and non-governmental entities to stem PO-related problems have been implemented since 2010.

**Findings:**

We compared the prevalence of NMPOU in the Ontario general adult population (18 years+) in 2010 and 2011 based on data from the ‘Centre for Addiction and Mental Health (CAMH) Monitor’ (CM), a long-standing annual telephone interview-based representative population survey of substance use and health indicators. While ‘any PO use’ (in past year) changed non-significantly from 26.6% to 23.9% (Chi2 = 2.511; df = 1; p =  0.113), NMPOU decreased significantly from 7.7% to 4.0% (Chi2 = 14.786; df = 1; p < 0.001) between 2010 and 2011. Over-time changes varied by age group but not by sex.

**Conclusions:**

The observed substantial decrease in NMPOU in the Ontario adult population could be related to recent policy interventions, alongside extensive media reporting, focusing on NMPOU and PO-related harms, and may mean that these interventions have shown initial effects. However, other casual factors could have been involved. Thus, it is necessary to systematically examine whether the observed changes will be sustained, and whether other key PO-related harm indicators (e.g., treatment admissions, accidental mortality) change correspondingly in order to more systematically assess the impact of the policy measures.

## Background

Since about 2000, non-medical use and harms (e.g., morbidity, mortality) related to prescription opioids (POs) have emerged as a major substance use and public health problem in North America, including Canada [1,2]. While key facets of this very problem are solidly documented in the United States (US), the only country in the world with a higher overall PO consumption (in Defined Daily Doses, DDD) per capita than Canada, relevant indicator data for Canada have been available only selectively, in part due to inadequate monitoring systems [1,3,4].

The most comprehensive Canadian data on NMPOU and PO-related harms come from Ontario, its most populous province. There are two reasons for this: overall PO consumption rates (a known determinant of levels of PO-related harms) are higher in Ontario than in most other provinces, and it has the most comprehensive monitoring data available [5,6]. Specifically, recent Ontario data have indicated high levels of non-medical prescription opioid use (NMPOU) in general adult (5.9%; 2010/11) and secondary student (15.5%; 2011), as well as key marginalized (e.g., aboriginal on reserves, street drug users) populations [1,7,8]. Furthermore, there have been substantive increases in PO-related substance use treatment admissions and increases in PO-related accidental mortality since about 2000 [1,2,9]. In addition, extensive variations of PO prescribing, including possible over-prescribing, associated with elevated morbidity and mortality risks have been documented in the Ontario population [10,11].

While Canada, unlike countries like the US or Australia, yet remains without a national strategy targeting the problem of NMPOU and PO-related harms, various governmental and non-governmental entities in Ontario have implemented a number of large-scale interventions aiming to reduce these problem phenomena in the past couple of years. For example, in 2010, the Ontario College of Physicians and Surgeons (CPSO) issued a report containing numerous recommendations for improved regulatory control, clinical practice guidelines, and interventions related to POs with the aim of reducing the “opioid public health crisis” in Ontario [12]. In November 2010, the Ontario government passed the ‘Narcotics Safety and Awareness Act’ (NSAA) as the legal foundation of its new provincial ‘Narcotics Strategy’ launched in 2011 [13]. A core feature of the NSAA was the implementation of a prescription monitoring program (PMP) centrally collecting information on POs dispensed by prescribers and patients [13]. PMPs have been in place in most US states and Canadian provinces (but not Ontario) and are associated with reduced PO dispensing levels, albeit their effects on PO abuse or harms are not clearly evidenced [14,15]. In early 2012, the province delisted ‘Oxycontin’ (oxycodone) – a potent PO substance associated with an extensive share of NMPOU and PO-deaths—which accounted for 25% (in Defined Daily Doses per population) of PO dispensing in 2010 in Ontario [5] from the province’s formulary, triggering a substantive reduction of its clinical use and availability [16]. These initiatives have been accompanied by extensive media reporting on the extent of NMPOU and PO-related harms effects on individual and public health [17,18].

The above interventions mostly occurred on an ad-hoc basis, and commentators have emphasized the need for systematic evaluations of their effects on reducing NMPOU and harms in Ontario, in the interest of science, evidence-based policy and public health [16,19]. Important markers for such evaluative efforts are indicators of both any PO use and NMPOU among adults. These specific indicators are assessed for Ontario by the ‘Centre for Addiction and Mental Health (CAMH) Monitor’ (CM), a long-standing survey on substance use and health indicators of the Ontario general adult population.

## Methods and findings

Relevant annual cycles of the CM were based on telephone interviews with randomly selected annual samples of n = 2024 (2010) and n = 1999 (2011; response rate 51% for both years) adults ages 18 and older, representative of the Ontario general adult population. Specifically, ‘any PO use’ in the past year was assessed by the combined results of the two questions: “In the past 12 months how many times, if at all, have you used any pain relievers (such as Percocet, Percodan, Tylenol #3, Demerol, OxyContin, codeine) with a prescription or because a doctor told you to take them?” and “In the last 12 months, if at all, how often did you use pain relievers without a prescription or without a doctor telling you to take them?”; NMPOU was assessed by the second question item only [for further CM methods details, see [20]].

While ‘any PO use’ in the past year changed non-significantly (26.6% and 23.9%; Chi2 =2.51; df = 1; p = 0.113; all comparisons were conducted by Rao-Scott adjusted Chi2 test with complex survey design weighting), the overall rate of NMPOU decreased significantly from 7.7% to 4.0% (Chi2 =14.79; df = 1; p < 0.001) among Ontario adults from 2010 to 2011. Examining the changes by subgroups, there was a significant interaction of survey year with age (adjusted Wald test, F (2, 3907) = 3.62; p = 0.027): reductions occurred among age groups 55+ years (7.6% to 2.1%; Chi2 = 30.41; df = 1; p < 0.001) and 30 – 54 years (8.1% to 3.6%; Chi2 = 9.98; df = 1; p = 0.002) but not among ages 18 – 29 years (7.0% to 7.0%; Chi2 = 0.00; df = 1; p = 0.987). With respect to sex, the interaction was only marginally significant at the 7% level (adjusted Wald test, F (1, 3996) = 3.45; p = 0.063), indicating that these data offered no evidence that the reductions in NMPOU were more pronounced in women than in men.

## Conclusions

One possible explanation for the observed substantial (almost 50%) recent decrease in NMPOU among Ontario adults could be that recent policy measures, combined with extensive media coverage and evolving social debate focusing on PO abuse and related harms in Ontario, may have had some initial effects. However, there is no empirical evidence for such causal influence at present which would, for example, require time-series analyses of relevant indicators if available. Alternatively, factors like ecological or natural changes in general awareness about NMPOU, PO-related drug use behaviors or availability could have contributed to the observed NMPOU reductions. The demonstrated sizeable drop in NMPOU among Ontario adults mirrors recent developments in the US. There, NMPOU has declined in the general adult population as measured by the National Survey of Drug Use and Health (NSDUH); specifically, NMPOU (in past year) among adults 18+ years has declined significantly from 4.7% (2010) to 4.2% (2011; p < 0.01) [21]. The parallel reductions in NMPOU in the US might suggest natural or more universal declines in NMPOU; however, the US has also seen a variety of targeted interventions and media reporting regarding NMPOU and PO-related harms that may have influenced these phenomena.

Notably, the changes in NMPOU observed in Ontario are markedly uneven by socio-demographic subgroups, i.e. sex and age. This deviates from previously observed patterns of a rather even spread of NMPOU across these socio-demographic groups [7]. Importantly, the above NMPOU indicators should be examined over a longer period of time, and other indicators need to be monitored, in order to assess whether substantive reductions in NMPOU are sustained, and other PO-related harms are correspondingly reduced in Ontario. For example, NMPOU has been reported to be considerably higher among Ontario secondary students than in adults, where it is however not evident at this point whether similar reduction effects are occurring [7,8]. Furthermore, PO-related emergency room episodes, substance use treatment admissions as well as accidental poisoning are all essential harm indicators which need to be assessed in order to determine whether PO-related burden of disease has actually been reduced [4,22]. In addition to these, it will be equally important to monitor pain care quality, and availability, as these could be compromised as an unintended consequence of the above interventions focusing on PO use [23]. While such evaluative future monitoring efforts are reasonably feasible with existing data in Ontario, the fragmented existence (or absence) of systematically comparable indicators across Canada unfortunately hinders important comparative evaluation efforts [1].

While it is yet uncertain whether the observed reductions in NMPOU are causally related to the described recent interventions, and whether other key PO-related problems in Ontario are characterized by corresponding decreases, there are examples from other jurisdictions where such reductions have been observed following targeted interventions. For example, the recent implementation of more restrictive PO dosing guidelines has resulted in substantive reduction of PO-related mortality in Washington State [24]. The burden of disease stemming from NMPOU and harms is extensive in Ontario [25], and it is essential from both science and policy perspectives to know whether the comprehensive interventions launched have been effective or not.

## Competing interests

The authors declare that they have no competing interests.

## Authors’ contributions

BF led the overall study and writing; RM, JR and AI co-led the CM survey and data analyses. All authors contributed substantively to data analyses and interpretation, as well as contributed to manuscript drafting and revisions. All authors read and approved the final manuscript.
